# Supplementary material for: Empirical methods for controlling false positives and estimating confidence in ChIP-Seq peaks
Source: BMC Bioinformatics. 2008 Dec 5;9:523. doi: 10.1186/1471-2105-9-523 (PMC2628906; doi:10.1186/1471-2105-9-523)
Supplement: Additional File 1 — A variety of html documents from the USeq web site detailing the available applications, their best usage, output file type descriptions, command line menus, etc. [file 1471-2105-9-523-S1.zip › USeqUserGuides/JavaDoc.zip!/JavaDoc/util/bio/xamer/XamerCounter.html]

XamerCounter


|  |  |  |
| --- | --- | --- |
| **Overview** \| **Package** \| **Class** \| **Use** \| **Tree** \| **Deprecated** \| **Index** \| **Help** | |  |
| **PREV CLASS**   NEXT CLASS | **FRAMES**    **NO FRAMES**     **All Classes** |  |
| SUMMARY: NESTED \| FIELD \| CONSTR \| METHOD | DETAIL: FIELD \| CONSTR \| METHOD |  |


---


## util.bio.xamer Class XamerCounter

```
java.lang.Object
  util.bio.xamer.XamerCounter
```

---

``` public class XamerCounter extends java.lang.Object ```

Counts the frequency of random words in a list of sequences.

---

**Constructor Summary**

`XamerCounter()`


| **Method Summary** | |
| --- | --- |
| `int[]` | `calculateTotalNumberOfOligosPerRound(java.lang.String[][] seqs)` |
| `static java.lang.String[][]` | `fetchSequences(java.lang.String[] files)`             Fetch all sequences from each multi-FASTA file |
| `static java.util.ArrayList` | `findSequenceHits(java.lang.String[] seqs, java.lang.String oligo)`             Looks for an exact match of the oligo to each of the seqs, returning the seqs that contain the oligo |
| `static java.lang.String[]` | `hexamerGenerator()` |
| `static void` | `main(java.lang.String[] args)` |
| `static java.lang.String[]` | `pentamerGenerator()` |
| `static java.lang.String` | `reverseCompDNA(java.lang.String seq)` |
| `static java.util.ArrayList` | `searchForXamers(java.lang.String[] xamers, java.lang.String[][] seqs)`             Creates an ArrayList of Xamer objects, where each Xamer contains all the seqs, by round that were found to match a particular xamers[i] |
| `void` | `setRevCompRefs(java.util.ArrayList xamers)`             Set refs to reverse comp xamer |

**Methods inherited from class java.lang.Object**

`equals, getClass, hashCode, notify, notifyAll, toString, wait, wait, wait`

**Constructor Detail**

### XamerCounter

```
public XamerCounter()
```


**Method Detail**

### setRevCompRefs

```
public void setRevCompRefs(java.util.ArrayList xamers)
```

:   Set refs to reverse comp xamer

---


### reverseCompDNA

```
public static java.lang.String reverseCompDNA(java.lang.String seq)
```

---


### searchForXamers

```
public static java.util.ArrayList searchForXamers(java.lang.String[] xamers,
                                                  java.lang.String[][] seqs)
```

:   Creates an ArrayList of Xamer objects, where each Xamer contains all the seqs, by round
    that were found to match a particular xamers[i]

---


### calculateTotalNumberOfOligosPerRound

```
public int[] calculateTotalNumberOfOligosPerRound(java.lang.String[][] seqs)
```

---


### findSequenceHits

```
public static java.util.ArrayList findSequenceHits(java.lang.String[] seqs,
                                                   java.lang.String oligo)
```

:   Looks for an exact match of the oligo to each of the seqs, returning the seqs that contain
    the oligo

---


### fetchSequences

```
public static java.lang.String[][] fetchSequences(java.lang.String[] files)
```

:   Fetch all sequences from each multi-FASTA file

---


### main

```
public static void main(java.lang.String[] args)
```

---


### hexamerGenerator

```
public static java.lang.String[] hexamerGenerator()
```

---


### pentamerGenerator

```
public static java.lang.String[] pentamerGenerator()
```


---


|  |  |  |
| --- | --- | --- |
| **Overview** \| **Package** \| **Class** \| **Use** \| **Tree** \| **Deprecated** \| **Index** \| **Help** | |  |
| **PREV CLASS**   NEXT CLASS | **FRAMES**    **NO FRAMES**     **All Classes** |  |
| SUMMARY: NESTED \| FIELD \| CONSTR \| METHOD | DETAIL: FIELD \| CONSTR \| METHOD |  |


---
